# Child Care Provider Training and a Supportive Feeding Environment in Child Care Settings in 4 States, 2003

**Published:** 2011-08-15

**Authors:** Madeleine Sigman-Grant, Elizabeth Christiansen, George Fernandez, Janice Fletcher, Laurel Branen, Beth A. Price, Susan L. Johnson

**Affiliations:** University of Nevada Cooperative Extension; University of Nevada, Reno, Nevada; University of Nevada, Reno, Nevada; University of Idaho, Moscow, Idaho; University of Idaho, Moscow, Idaho; University of Idaho, Moscow, Idaho; University of Colorado Denver, Anschutz Medical Campus, Aurora, Colorado

## Abstract

**Introduction:**

Strategies to prevent adult chronic diseases, including obesity, must start in childhood. Because many preschool-aged children spend mealtimes in child care facilities, staff should be taught supportive feeding practices for childhood obesity prevention. Higher obesity rates among low-income children suggest that centers providing care to these children require special attention. We compared self-reported feeding practices at child care centers serving low-income children on the basis of whether they received funding and support from the Child and Adult Care Food Program (CACFP), which suggests supportive feeding practices. We also assessed training factors that could account for differences among centers.

**Methods:**

Eligible licensed child care centers (n = 1600) from California, Colorado, Idaho, and Nevada received surveys. Of the 568 responding centers, 203 enrolled low-income families and served meals. We analyzed the responses of 93 directors and 278 staff for CACFP-funded centers and 110 directors and 289 staff from nonfunded centers. Chi square analyses, pairwise comparisons, *t* tests, and multiple linear regressions were used to compare CACFP-funded and nonfunded centers.

**Results:**

Significant differences were noted in 10 of 26 feeding practices between CACFP-funded and nonfunded centers. In each case, CACFP-funded centers reported practices more consistent with a supportive feeding environment. Forty-one percent of the variance could be explained by training factors, including who was trained, the credentials of those providing training, and the type of training.

**Conclusion:**

Our findings suggest that when trained by nutrition professionals, child care staff learn, adopt, and operationalize childhood obesity prevention feeding guidelines, thereby creating a supportive mealtime feeding environment.

## Introduction

Strategies to encourage healthy eating and active living are needed to prevent childhood obesity (1). Although the family is primarily responsible for the health habits of children aged 2 to 5, many preschool-aged children (including those from low-income families) spend a substantial amount of time in child care facilities, making these settings appropriate for initiating obesity prevention practices (2-6). Best practices include creating a supportive mealtime environment that allows children control over their food intake in response to internal cues (1,7,8) and implementing Satter's Division of Feeding Responsibility (9,10). Satter's widely accepted concept postulates a distribution of feeding and eating roles between child and adult. Adult caregivers are responsible for selecting, preparing, and offering healthful, appropriate foods and determining when and where food is served. Children are responsible for how much of these foods they eat and whether they eat at all.

**Box. Components of a Supportive Feeding Environment^a^**

**1) Physical**

Size and type of forks, spoons, knives, pitchers, cups, and serving utensils (D)

Type of food service (DS)

**2) Social**

Teaching at mealtimes (S)

Language (D)

Role modeling (DS)

**3) Developmental**

Self-feeding skills (S)

Serving skills (S)

Trying new foods (S)

Teaching at mealtimes (S)

**4) Established routines**

Timing of meals (D)

Staff roles at mealtimes (DS)

Teaching at mealtimes (S)

Conversations (S)

Serving style (DS)

**5) Trust**

Responding to children’s hunger and fullness cues (S)

Allow children to eat according to internal cues of fullness and hunger (S)

Abbreviations: D, responses by directors; S, responses by staff; DS, responses by directors and staff for similarly constructed questions.aThese 5 components reflect the concepts that define a supportive mealtime environment. They are derived from the 2003 About Feeding Children staff and director questionnaires (11,12). Several components appear under multiple headings.

To our knowledge, no published studies define or validate what constitutes a supportive feeding environment. Hence, for the purposes of this study, we define a supportive feeding environment as one that offers children adequate nutritious food in a physical and social setting that enhances their development, establishes routines, and maintains trust (11,12) (Box). In this environment, adults use feeding practices that help children to eat according to their hunger and fullness cues. Physical needs are met with appropriate space, equipment, and utensils. Social components are sharing, togetherness, conversation, and listening. To promote self-help skills, encouraging, friendly staff model healthy eating, serve meals at consistent times and locations, and help children feed and serve themselves and respond to internal cues (13).

Serving appropriate food in amounts consistent with growth and promoting increased physical activity are the cornerstones of preschool childhood obesity prevention at any income level. As a US Department of Agriculture (USDA) supplemental nutrition assistance program, the Child and Adult Care Food Program (CACFP) provides meals and snacks to 3.2 million low-income American children daily (14) through eligible entities (for eligibility requirements see www.fns.usda.gov/cnd/care/CACFP/aboutcacfp.htm). By specifying types of foods and serving sizes (14-16), CACFP has a positive effect on children's nutrient intake (16,17). For more than 10 years, CACFP has suggested feeding strategies that are congruent with messages aimed at reducing childhood obesity (1,7,8,13,18,19). CACFP requires yearly training for participating staff and agencies, although these trainings focus more on program integrity issues than on feeding guidance. Although CACFP does not address physical activity, the program's written feeding suggestions deal with a clean and safe setting, family-style service (defined at the time of this study as allowing children to serve themselves from common bowls and dishes), preparing enough food to meet the needs of all enrolled children, and allowing seconds. However, except for Head Start, CACFP-funded centers are not required to follow these suggestions. Frisvold credits Head Start nutrition services and nutrition education as being responsible for the significantly lower rate of later childhood obesity in black children who participated in Head Start during their preschool years (20). Although not addressed in Frisvold's study, both Satter's Division of Feeding Responsibility and the family-style meal service mandated by some states for Head Start also might have contributed. Because children from low-income households are at higher risk for obesity (1), CACFP-funded centers could play an important role in modulating the prevalence of childhood obesity (4). Given that licensing agencies in most states do not require specific feeding practices, it is unlikely that nonfunded centers serving low-income families would adhere to a formal set of supportive feeding practices. The objective of this study was to compare CACFP-funded to similar nonfunded centers in terms of staff's and directors' reported mealtime feeding practices. In addition, we explored center and staff training in these practices.

## Methods

### Data source

This report is based on results from the 2003 About Feeding Children Study (11), funded by USDA. Licensed child care centers (n = 1,600; 8,000 staff and 1,600 directors) from 4 Western states (California, Colorado, Idaho, Nevada) serving children aged 18 to 60 months were eligible for inclusion. The centers were randomly stratified by location because not all licensing agencies could provide information regarding staffing, meal service, family income, or children's ages. For every center, 1 administrator (defined as anyone who provided center oversight such as director, owner, or supervisor) and up to 5 staff members could respond individually. Directors and staff specified their multiple roles at the center (eg, owner and director, assistant teacher and cook). A 60-question staff questionnaire queried staff feeding behaviors, attitudes, beliefs, practices, training, and demographic characteristics. A 30-question director questionnaire queried center-specific details about feeding policies, training, income level of families served, and director demographic characteristics. The institutional review boards (IRBs) from the universities of Nevada, Idaho, and Colorado approved all protocols. (California child care centers were included in the University of Nevada IRB approval.) The adjusted overall response rate was 41%, representing responses from 568 centers, 1,190 staff, and 464 directors. Further details regarding sampling, questionnaire design, validation, distribution, and response rates are reported elsewhere (11). For the purposes of this study, we included only centers self-identifying as serving low-income children, grouped into CACFP-funded and nonfunded categories.

We designed both the director and staff questionnaires to elicit responses about feeding practices and mealtime environments, specifically to determine how supportive mealtime suggestions were operationalized. For example, we asked both the director and staff about the type of meal service provided (eg, family-style, lunch bags, preplated). We asked staff about 1) behaviors that would describe supportive meal environments (eg, whether children have a chance to make decisions about what to eat and the order in which food should be eaten, whether they are allowed to choose how much to eat without adult interference), 2) whether children could practice serving themselves in an environment that provided adult guidance, 3) whether staff sat at the table and what they did during mealtimes, and 4) staff training in child feeding. We asked directors about 1) whether staff were required to sit with the children during mealtime (a practice that allows staff to serve as role models and to monitor behavior and assist children), 2) what equipment (eg, cups, forks) was available, and 3) who conducted child feeding training for directors and staff and the extent and type of training. We performed construct and content validity on both questionnaires by triangulating 10 expert panel members’ input and review, separate structured interviews of 49 staff and 12 directors, and assessment by a survey design expert.

Of the 568 responding centers, 346 enrolled low-income families; however only 203 centers served meals. Of these, 93 centers received CACFP funding. Hence, analyses were conducted on responses of 93 directors and 278 staff for CACFP-funded centers and 110 directors and 289 staff of nonfunded centers.

### Statistical analysis

We used SPSS version 6.1 (SPSS, Inc, Chicago, Illinois) for database management and SUDAAN version 9.0.1 (RTI International, Research Triangle Park, North Carolina) for all statistical analyses to account for the complex sampling design (21). We used the Taylor series linearization variance estimation method in SUDAAN to compute the corrected standard errors. We conducted χ^2^ analyses; results were considered significant at *P* < .05. For items with more than 2 response options, we further examined significant associations to determine the response options for which significant differences between CACFP-funded and nonfunded centers or staff occurred. Pairwise comparison differences were considered significant at *P* < .05. We used *t* tests to compare means of continuous variables and composite variables (derived by summing responses from several variables) for significant differences.

We constructed a final composite variable using 26 survey items that related to a supportive feeding environment in child care centers (Appendix), chosen on the basis of suggested child obesity prevention messages in the literature and Satter's Division of Responsibility. Because items had different response options, they were standardized before being combined into the index variable. Cronbach α measuring internal reliability was acceptable (α = .73) (22). Multiple linear regression analysis was conducted to determine which training items contributed significantly to the prediction of a supportive feeding environment, regardless of CACFP status, by using SAS version 9.1 (SAS Institute, Inc, Cary, North Carolina). Child feeding training items included type of director training, who provided training to center staff, frequency with which director sought information or participated in professional development, and frequency of center cooks' training.

## Results

### Demographic characteristics

Of centers receiving CACFP funding, 23 (25%) were Head Start centers. More white, non-Hispanic staff worked in nonfunded centers (mean [standard error (SE)], 75% [2.9 %] vs 41% [2.4%]; *t* = −4.01; *P* = .001). Nearly all respondents were women. Forty-four percent of all staff reported having a college degree or some college education. There was no difference in age, years of child care experience, or years at the current center between staff from CACFP-funded and nonfunded centers. Moreover, there was no difference in staff participation in child feeding training by funding source (49% CACFP-funded vs 46% nonfunded).

### Supportive feeding environment practices

We found significant differences between CACFP-funded and nonfunded centers for 10 of 26 feeding practices (Table 1). In each case, CACFP-funded centers reported practices more consistent with a supportive feeding environment than did nonfunded centers. A higher proportion of CACFP-funded staff allowed children involvement in determining what to eat, the order in which to eat, and how much to eat, and a higher proportion of CACFP-funded centers used family-style meal service.

Most respondents from both CACFP-funded and nonfunded centers noticed and commented on (90%), and praised (95%) children who were eating well, commented on other children who were eating well (58%), and encouraged children to eat at least 1 bite of each food (63%). Most also reported never using food as a way to get children to do something (87%) or for consoling children when they were sad (87%). Finally, 27% strongly disagreed with the statement, "Adults know better than children how much children need to eat."

No significant differences were noted between CACFP-funded and nonfunded centers in terms of teaching specific skills to children (Table 2). Moreover, no differences were detected between CACFP-funded and nonfunded centers in caregivers' use of certain strategies to get children to eat new foods. Strategies included role modeling, teaching, coaxing, and restriction. Caregivers concurred with predicted effectiveness of these strategies; at least two-thirds reported that they believed each strategy would work.

### Training about feeding children

Less than 24% of staff from both CACFP-funded and nonfunded centers received more than yearly training about feeding children; however, differences were noted between cooks and administrators (Table 3). Furthermore, centers did differ in terms of who provided training. More than three-fourths of directors or site supervisors provided some training for both groups; however, owners of CACFP-funded centers (15%; SE, 1.0%) trained staff less often than owners of nonfunded centers (37%; SE, 4.5%) (*F* = 15.34, *P* = .03). Approximately one-third of the CACFP centers relied on the CACFP monitor, the person responsible for ensuring compliance with CACFP regulations and policies, to conduct some portion of the training. Moreover, significantly more CACFP-funded center staff were trained by nutrition professionals than were nonfunded center staff (12% [SE, 1.3%] vs 1% [SE, 0.1%] by registered dietitians [F = 8.02, *P* = .01] and 33% [SE, 1.8%] vs 13% [SE, 1.9%] by nutrition specialists [*F* = 9.34; *P* = .046]). Although 24% of directors from all centers reported being trained by health departments, differences were noted in the types of materials used for training between both staff and directors.

Among the training items significantly associated with a supportive feeding environment, 10 were positively associated and 7 were negatively associated (Table 4). In the final model 41% of the variance in supportive feeding environment was explained by these independent variables (*F*_18,353_ = 14.56, *P* < .001).

## Discussion

Although directors and staff in CACFP-funded centers were significantly more likely than those in nonfunded centers to engage in some supportive feeding practices, not all CACFP-funded centers trained staff in this area. In addition, not all trained staff reported appropriate supportive feeding practices. These findings reveal an important consideration when discussing strategies to prevent obesity in low-income children: lack of training of child care staff about feeding children. If child care centers are to engage in obesity prevention (1,7,8), anyone involved with feeding children must be trained. Feeding practices that support healthy weight should be stressed, whereas practices that could increase the risk of obesity should be discouraged. The latter include restrictive practices, such as "no sweets before you finish your meal," improper use of praise and attention to "good eaters," and strategies to get children to eat what providers believe they should eat. Goodell et al noted that Head Start caregivers' attitudes influenced what they expected a child to consume (23); child size was a factor in how much they served. Overweight children still need access to adequate food and nutrients for proper growth and development (15,24). Without appropriate training to guide caregivers in working with overweight children, unintentional but serious consequences may result, such as inadvertently restricting access to nutritious food while trying to prevent obesity.

It is difficult to prove the effect of allowing children to self-serve on prevention of obesity (25), but evidence suggests that children who serve themselves waste less food and eat as much as 25% less than those for whom food was plated (26,27). Furthermore, when preschool-aged children were served portions double their age-appropriate size, they consumed 25% more of the entrée and increased their energy intake by 15%, compared with when they were served age-appropriate portions (26). Alternative strategies for helping children meet their hunger and satiety needs must be created for centers where preplated food service is used or where only the minimum amount of food is prepared (24).

Whereas overall training about feeding is necessary, our study suggests specific factors should be included when educating child care staff. Frequency of training may not be as important as where the information comes from and who provides it, that is, the orientation and education of the trainer. Information provided by perceived credible sources and people qualified to teach nutrition results in positive practices. Hence, we were surprised that training provided by health departments was negatively associated with practice. We speculate that health department training has more to do with environmental safety than with feeding children or that the training may be delivered by noncredentialed paraprofessionals who do not have expertise in feeding young children. Indeed, some public health department policies prohibit supportive feeding strategies such as allowing children to serve themselves from a common bowl or to participate in food preparation.

This study was limited in geographic scope, preventing generalizability to other states. Despite this limitation, our findings suggest that when trained by nutrition professionals, child care staff can learn, adopt, and operationalize guidelines for a supportive feeding environment in preventing child obesity.

We were disheartened by the number of CACFP-funded centers where staff were not trained in child feeding. The lack of training may reflect the lack of designated CACFP funding set aside for training beyond that addressing compliance and integrity. The required training is often received by the cook or director, rather than teachers. Annual mandatory training for all involved with child feeding could increase knowledge about nutrition and child development, influence caregiver attitudes about feeding, and promote positive practices. CACFP training in child feeding, either in-person, online, or via written materials, should be available to and required for center directors, staff (including cooks), and anyone in the room at mealtimes. Much of the training regarding feeding occurs during site reviews; however, required center audits are conducted a minimum of only once every 3 years and include limited center personnel (oral communication, D. Hogan, MS, RD, CACFP Programs Professional, Nevada Department of Education). CACFP monitors can implement the use of assessment tools (12,28) during site visits and train centers to use these tools to improve feeding practices. External trainers and CACFP monitors need to design creative interventions to increase awareness of the role of self-service (and other feeding skills) in helping children monitor their energy needs and maintain a reasonable, healthy weight.

Findings from this study should be discussed with USDA and others concerned with the influence of CACFP in preventing child obesity. CACFP can serve as a model in developing healthy eaters and preventing childhood obesity. Nutrition regulations that include attention to feeding environments and nutrition standards should be established (4,15). Specific training funds are necessary to increase awareness, demonstrate role modeling, change perceptions, facilitate acceptance of appropriate feeding strategies, and encourage use of self-assessment tools. Additionally, any center eligible for CACFP services should be encouraged to enroll. The advantage to enrolling in CACFP goes beyond reimbursement for food; it can provide exposure to, and support of, education and training to prevent childhood obesity.

## Figures and Tables

**Table 1:** Prevalence of Supportive and Nonsupportive Practices to Prevent Childhood Obesity Among Child Care Centers in 4 States (n = 203), by Funding Source, 2003^a^

Practice	CACFP-Funded, % (SE)	**Nonfunded,** % (SE)	Statistic^b^	*P* Value
**Supportive**				
Use family-style meal service^c^	93 (2)	45 (9)	*F* = 91.41	<.001
Sit at the table with children^d^	75 (6)	50 (7)	*t* = 2.54	.01
Provide children with child-size pitchers^c^	73 (7)	45 (8)	*F* = 171.30	.01
Talk about the food at mealtimes^d^	95 (2)	83 (5)	*t* = 3.02	.03
Strongly disagree that if children put food on their plate, they should eat it^d^	35 (7)	14 (4)	*t* = 2.51	.01
**Nonsupportive**				
Have children finish their meal before eating sweet foods^d^	36 (6)	71 (5)	*F* = 18.64	<.001
Have children eat nutritious foods before "junk foods"^d^	66 (5)	91 (3)	*F* = 22.54	<.001
Always have children finish healthy foods before they eat sweet foods^d^	19 (5)	43 (5)	*t* = −3.35	.001
Do not teach anything at mealtimes^d^	2 (0.9)	9 (9)	*t* = −2.45	.02
Often encourage children to eat the amount of food they think children need^d^	13 (3)	24 (3)	*t* = −2.37	.02

Abbreviations: CACFP, Child and Adult Care Food Program; SE, standard error.

a Based on returned surveys of 93 directors and 278 staff from 93 CACFP-funded centers and 110 directors and 289 staff from 110 nonfunded centers to a randomized survey of child care centers serving low-income children in California, Colorado, Idaho, and Nevada (11). Not all directors or staff responded to every item.

b The *t* value is reported for pairwise comparisons and only for items with multiple response options instead of binary options. The *F* value is reported for items with binary response options.

c Indicates responses given by directors.

d Indicates responses given by staff.

**Table 2:** Use and Expected Outcomes of Selected Strategies to Get Children to Eat New Foods Among Child Care Staff in 4 States (n = 203), 2003^a^

**Strategy**	Use, %	Belief in Efficacy, %
Asking child to take a bite	94	92
Trying food with children	93	98
Teaching about food before serving	86	97
Withholding sweet foods until food is tried	46	79
No seconds unless a food is tried	25	67

a Based on returned surveys from 567 staff from 203 centers to a randomized survey of child care centers serving low-income children in California, Colorado, Idaho, and Nevada (11). Not all staff responded to every item.

**Table 3:** Differences in Child Feeding Training Characteristics Among Child Care Centers in 4 States (n = 203), by Funding Source, 2003^a^

Characteristic	CACFP-Funded, % (SE)	**Nonfunded, % (SE)**	Statistic^b^	*P* Value
More than annual training for administrators and supervisors	36 (2.3)	13 (1.6)	*t* = 2.31	.02
More than annual training for cooks	50 (2.9)	17 (1.4)	*t* = 2.98	.003
Director trained on site	65 (2.1)	22 (4.1)	*F* = 96.92	<.001
Use of USDA materials	88 (0.9)	20 (2.0)	*F* = 23.45	<.001
New staff attend workshop or seminar	13 (0.9)	0.5 (0.04)	*F* = 418.21	.03
New staff view training tapes	13 (0.9)	0.4 (0.02)	*F* = 415.69	.03

Abbreviations: CACFP, Child and Adult Care Food Program; SE, standard error.

a Based on returned surveys from 93 directors of CACFP-funded and 110 directors of nonfunded centers to a randomized survey of child care centers serving low-income children in California, Colorado, Idaho, and Nevada (11). Not all directors responded to every item.

b The *t* value is reported for pairwise comparisons and only for items with multiple response options instead of binary options. The *F* value is reported for items with binary response options.

**Table 4:** Presence of a Supportive Feeding Environment, by Characteristics of Training About Feeding Children, Among Child Care Centers in 4 States (n = 145), 2003^a^

**Characteristic**	β (SE)	*P* Value
**Type of training director has had about feeding children**		
Materials or trainings through CACFP or state food program	11.00 (3.10)	<.001
Information provided by Cooperative Extension	8.73 (2.26)	<.001
Reading popular books and magazines	6.99 (1.77)	<.001
Information provided by a nutritionist or health consultant	6.66 (1.83)	<.001
Center trainings (on site)	4.02 (1.83)	.03
Information provided by health department	−4.23 (1.75)	.02
Training on how to use specific classroom curriculum such as Chef Combo or Food Groupies	−4.90 (2.50)	.05
Reading newsletters or brochures	−8.83 (2.05)	<.001
Workshops or conferences	−9.94 (2.35)	<.001
**Person who provided training to staff**		
Director or site supervisor	9.77 (1.92)	<.001
Registered dietitian	5.85 (2.13)	.01
Nutrition specialist	5.78 (1.97)	<.001
Cook	−4.16 (2.04)	.04
Outside consultant/workshop presenter	−5.09 (1.86)	<.001
Teacher	−7.98 (2.18)	<.001
**How often director tries to become more informed or participate in professional development about feeding children**	4.74 (1.19)	<.001
**How often cooks receive training about feeding children**	1.46 (0.74)	.05

Abbreviation: SE, standard error.

a "Supportive feeding environment" is a composite variable constructed by using 26 items selected from a randomized survey of child care centers serving low-income children in California, Colorado, Idaho, and Nevada (11). Because the items had different response options, all items were standardized before being combined into the index variable. Surveys were returned by 567 staff and 203 directors from 203 centers; however, not all directors or staff responded to every item. Multiple linear regression analysis was conducted controlling for CACFP funding status (β = −0.19, SE = 2.88, *P* = .52).

## Appendix: A Supportive Mealtime Environment Index Based on Selected Questions Used in the About Feeding Children Study^a^

### Queried of staff

How often do you encourage children to eat the amount you think they need?^b^

How often do you require children to finish all the food on their plates?^b^

How often do you have children finish healthy foods before they eat sweet foods?^b^

How often do you use food as a way to get children to do things?^b^

How often do you offer food to children when they are sad?^b^

When a child is feeling sad, it's OK to offer a cracker to help the child feel better.^c^

Adults know better than children how much children need to eat.^c^

If children put food on their plates, they should eat it.^c^

Children are more likely to try a new food after they see me eat it.^c^

I have the children eat nutritious food before "junk" food.^d^

I notice and comment to the child who is eating well.^d^

I say something like, "Pat is eating green beans. Why don't you eat some?"^d^

I talk about food at mealtime.^d^

I praise children when they eat well.^d^

I have the children eat one bite of each food.^d^

I have the children finish their meal before eating sweet foods.^d^

Staff teach in different ways at mealtimes. Choose the statement that best describes how you teach at mealtime.^e^

Which of these statements best describes what you usually do at mealtime?^f^

### Queried of directors

Think about the way food is served in your center. Choose the one that best describes what is done for preschool children.^g^

Our centers provide the following child-sized mealtime equipment for the preschooler and older child: forks; spoons; knives; pitchers; serving utensils; cups and glasses.^h^

a Concepts derived from the 2003 About Feeding Children Study staff and director questionnaires (11). b Response choices: always, often, sometimes, not often, never. c Response choices: strongly agree, somewhat agree, don't agree or disagree, somewhat disagree, strongly disagree. d Response choices: yes, no. e Response choices: I plan ahead what I am going to teach at mealtime; I don't plan, teaching is a natural part of the mealtime; I don't teach anything at mealtime. f Response choices: I sit with the children; I am in the room but don't sit with the children; I get up and down during mealtime. g Response choices (excluding those pertaining to children bringing in lunch or going through a cafeteria line): I/teacher serve(s) the children's plates and cups from bowls/pitchers; the food is already on the children's plates when it comes from the kitchen or caterer; children serve themselves from common bowls and pitchers. h Choose all that apply.
